# Initial Clinical Experience with ^90^Y-FAPI-46 Radioligand Therapy for Advanced-Stage Solid Tumors: A Case Series of 9 Patients

**DOI:** 10.2967/jnumed.121.262468

**Published:** 2022-05

**Authors:** Justin Ferdinandus, Pedro Fragoso Costa, Lukas Kessler, Manuel Weber, Nader Hirmas, Karina Kostbade, Sebastian Bauer, Martin Schuler, Marit Ahrens, Hans-Ulrich Schildhaus, Christoph Rischpler, Hong Grafe, Jens T. Siveke, Ken Herrmann, Wolfgang P. Fendler, Rainer Hamacher

**Affiliations:** 1 Department of Nuclear Medicine, West German Cancer Center, University Hospital Essen, University of Duisburg-Essen, Essen, Germany, and Uital Essen, Essen, Germany;; 2 German Cancer Consortium (DKTK), Partner Site University Hospital Essen, and German Cancer Research Center (DKFZ), Essen, Germany;; 3 Department of Medical Oncology, West German Cancer Center, University Hospital Essen, University of Duisburg–Essen, Essen, Germany;; 4 Medical Clinic II, University Hospital Frankfurt, Frankfurt am Main, Germany;; 5 Department of Pathology, West German Cancer Center, University Hospital Essen, Essen, Germany;; 6 Bridge Institute of Experimental Tumor Therapy, West German Cancer Center, University Medicine Essen, Essen, Germany; and; 7 Division of Solid Tumor Translational Oncology, German Cancer Consortium (DKTK, Partner Site Essen) and German Cancer Research Center (DKFZ), Heidelberg, Germany

**Keywords:** FAPI, theranostics, fibroblast activation protein, solid tumors

## Abstract

Fibroblast activation protein (FAP) is overexpressed in several solid tumors and therefore represents an attractive target for radiotheranostic applications. Recent investigations demonstrated rapid and high uptake of small-molecule inhibitors of FAP (^68^Ga-FAPI-46) for PET imaging. Here, we report our initial experience of the feasibility and safety of ^90^Y-FAPI-46 for radioligand therapy of extensively pretreated patients with solid tumors. **Methods:** Patients were considered for ^90^Y-FAPI-46 therapy if they showed both an exhaustion of all approved therapies based on multidisciplinary tumor board decision, and high FAP expression, defined as SUV_max_ greater than or equal to 10 in more than 50% of all lesions. If tolerated, ^90^Y-FAPI-46 bremsstrahlung scintigraphy was performed after therapy to confirm systemic distribution and focal tumor uptake, and ^90^Y-FAPI-46 PET scans were performed at multiple time points to determine absorbed dose. Blood-based dosimetry was used to determine bone marrow absorbed dose. Adverse events were graded using Common Terminology Criteria for Adverse Events (version 5.0). **Results:** Nine patients either with metastatic soft-tissue or bone sarcoma (*n* = 6) or with pancreatic cancer (*n* = 3) were treated between June 2020 and March 2021. Patients received a median of 3.8 GBq (interquartile range [IQR], 3.25–5.40 GBq) for the first cycle, and 3 patients received subsequent cycles with a median of 7.4 GBq (IQR, 7.3–7.5 GBq). Posttreatment ^90^Y-FAPI-46 bremsstrahlung scintigraphy demonstrated sufficient ^90^Y-FAPI-46 uptake in tumor lesions in 7 of 9 patients (78%). Mean absorbed dose was 0.52 Gy/GBq (IQR, 0.41–0.65 Gy/GBq) in the kidney, 0.04 Gy/GBq (IQR, 0.03–0.06 Gy/GBq) in bone marrow, and less than 0.26 Gy/GBq in the lung and liver. Measured tumor lesions received up to 2.28 Gy/GBq (median, 1.28 Gy/GBq). New laboratory G3 or G4 toxicities were noted in 4 patients (44%, *n* = 2 patients with thrombocytopenia only, *n* = 2 patients with new onset of thrombocytopenia and anemia). Other G3 or G4 laboratory-based adverse events occurred in 2 patients or fewer. No acute toxicities attributed to ^90^Y-FAPI-46 were noted. Radiographic disease control was noted in 4 patients (50%). **Conclusion:** FAP-targeted radioligand therapy with ^90^Y-FAPI-46 was well tolerated, with a low rate of attributable adverse events. Low radiation doses to at-risk organs suggest feasibility of repeat cycles of ^90^Y-FAPI-46. We observed signs of tumor response, but further studies are warranted to determine efficacy and the toxicity profile in a larger cohort.

## Visual Abstract

**Figure:**
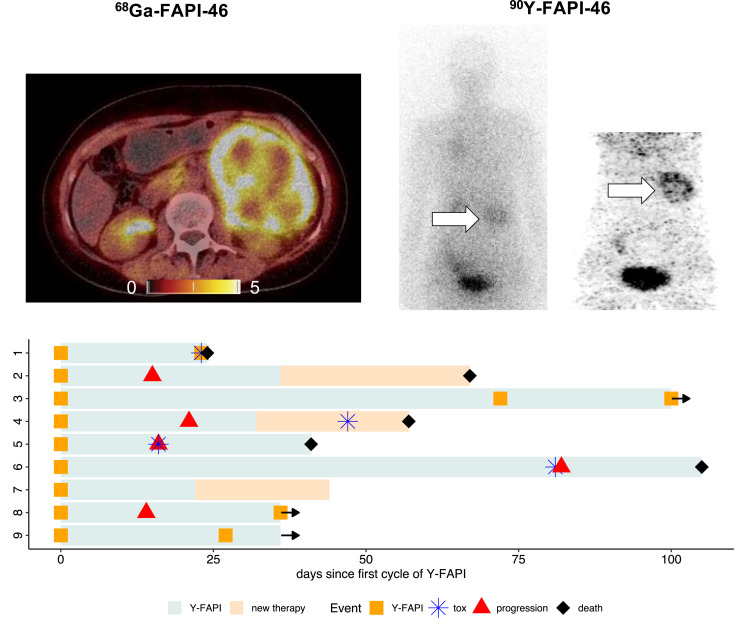

The fibroblast activation protein (FAP) is expressed by cancer-associated fibroblasts as well as cancer cells such as sarcoma and mesothelioma (1–3). Therefore, FAP is an attractive target for both imaging and radionuclide therapy of solid tumors. Previously, several groups have described high tumor uptake for ^68^Ga- or ^18^F-labeled PET compounds (4–9). For imaging, we used the FAP-targeted inhibitor FAPI-46 for diagnostic work-up of cancer types such as pancreatic cancer and sarcoma (10*,*^11^).

Recently, FAP-targeted radioligand therapy (RLT) has been described in several case reports (12–14); however, feasibility has not yet been systematically analyzed. In this case series, ^90^Y-labeled FAPI-46 (^90^Y-FAPI-46) RLT was offered to patients with advanced-stage solid tumors who have exhausted all established lines of treatment. ^90^Y features high-branching-ratio β^−^ emission (99.99%) with an endpoint energy of 2.280 MeV, allowing high dose deposition within defined tumor lesions. Its relatively short half-life of 64.1 h makes it appropriate for therapeutic combinations in which the biochemical vector exhibits a short target retention time. Preclinical studies on FAPI-46 have demonstrated a decrease to 30% of tumor uptake from 1 to 24 h after injection (14). Posttreatment ^90^Y-FAPI-46 scintigraphy is performed by measuring the β^−^ emission–associated bremsstrahlung radiation. ^90^Y decays by internal conversion (0.0032%), emitting a positron with a total kinetic energy of 0.760 MeV. Positron emission enables PET quantitative data for dosimetry (15).

In this study, we report on the safety, dosimetry, and response for repeat ^90^Y-FAPI-46 RLT in patients with advanced solid tumors.

## MATERIALS AND METHODS

**FIGURE 1.**
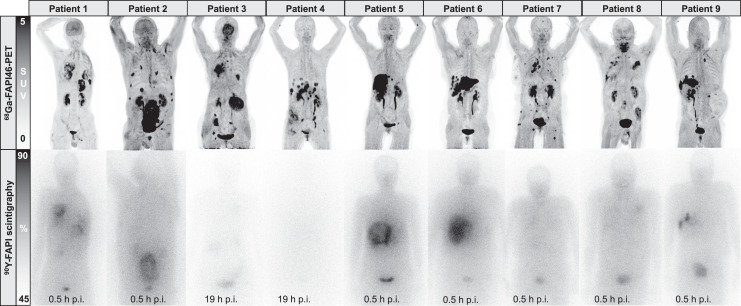
Pretreatment ^68^Ga-FAPI-46 PET images and posttreatment ^90^Y-FAPI-46 bremsstrahlung scintigraphs after first cycle of ^90^Y-FAPI-46 RLT. p.i. = after injection.

This was a monocentric, retrospective study of 9 patients with progressive, advanced-stage solid tumors receiving ^90^Y-FAPI-46 under compassionate access for a clinical indication. Radionuclide treatment was recommended by a multidisciplinary tumor board. All patients either had previously progressed during established treatment options or were not eligible to receive other treatments. This study was approved by the institutional review board (reference 21-9842-BO). All patients provided written informed consent to undergo clinical RLT and for retrospective analysis of clinical data. All patients underwent PET imaging with ^68^Ga-FAPI-46 before treatment to confirm the FAP positivity of tumor lesions, defined as an SUV_max_ greater than or equal to 10 in more than 50% of all lesions (Fig. 1). Imaging procedures were described previously (10); in brief, patients received a median of 103 MBq of ^68^Ga-FAPI-46 (interquartile range [IQR], 87–133.5 MBq) intravenously and were scanned at a median of 37 min (IQR, 24.5–60 min) after injection. To be eligible for treatment, patients needed adequate bone marrow function (i.e., leukocytes > 2.5/nL, hemoglobin > 7.0 mg/dL, and thrombocytes > 75/nL), with exceptions for patients receiving regular transfusions. Before treatment, renal scintigraphy with ^99m^Tc-MAG3 was performed to rule out urinary tract obstruction.

### ^90^Y-FAPI-46 Synthesis

^90^Y-FAPI-46 was synthesized using the Easyone synthesis module (Trasis) connected to shielded ^90^Y-YCl_3_ solution (Yttriga; Eckert and Ziegler). Before the automated synthesis started, the cassette was preloaded with FAPI-46 precursor (ABX, 8 μg/GBq), ascorbic acid, and sodium acetate buffer saline vials. The synthesis was fully automated using a good-manufacturing-practice–grade reagent and controlled by a preprogrammed sequence. The ^90^Y-YCl_3_ solution was transferred into the reactor, followed by the precursor and buffer mixture. For radiolabeling, the reaction mixture was heated to 90°C for 20 min. Afterward, the product was transferred into the bulk vial through a sterile filter and formulated with pentetic acid (1 mL, Ditripentat-Heyl; Heyl), ascorbic acid (∼40 mg/GBq, vitamin C; Rotexmedica), and saline. The quality control procedures included reverse-phase–high-performance liquid chromatography, instant thin-layer chromatography, pH, endotoxin, and sterility testing. The average yield was 88% ± 7%, reverse-phase–high-performance liquid chromatography radiochemical purity was 98% ± 1%, concentration was 883 ± 70 MBq/mL, and shelf life was 24 h.

### ^90^Y-FAPI-46 Administration

Patients underwent inpatient treatment to ensure radiation safety. Vital signs were monitored before and after administration of ^90^Y-FAPI-46. Patients 1 and 2 received a planned activity of 7.4 GBq of ^90^Y-FAPI-46 for the first cycle. All other patients received a planned first activity (scout dose) of 3.8 GBq of ^90^Y-FAPI-46 with dosimetry. Focal ^90^Y-FAPI-46 uptake was noted in more than 50% of tumor lesions on posttreatment ^90^Y-FAPI-46 bremsstrahlung scintigraphy (Fig. 1), and if clinically indicated, patients were eligible to receive further cycles with 2 doses of 3.8 GBq of ^90^Y-FAPI-46 (high dose), given on the same therapy day but 4 h apart. We chose fractionated applications to optimize prolonged radiation delivery on the basis of the observed short biologic half-life during scout cycles, which appeared to be less than 24 h. A therapeutic solution was administered intravenously with 500 mL of saline. Bremsstrahlung scintigraphy was performed approximately 24 h or, if possible, 0.5 h after therapy to confirm systemic distribution and focal tumor uptake. Whole-body planar imaging was performed at a scan speed of 10 cm/min, with an energy window of 90 − 125 keV and using a medium-energy collimator. All patients were discharged 48 h after administration, in accordance with radiation protection regulations.

### Dosimetry

If tolerated, patients underwent dosimetry after therapy. ^90^Y-FAPI-46 PET scans were not performed in cases of severe pain, a long acquisition (*n* = 3 during cycle 1 and *n* = 1 during cycle 2), or inability to tolerate or allow repeated blood sampling (*n* = 4). Bone marrow dosimetry was measured using repeated blood samples (0.5, 1, 2, 4, 24, 36, and 48 h after injection) and estimated according to OLINDA/MIRD recommendations. Dose absorbed by tumor lesions and kidneys was estimated using PET acquisitions. PET images were acquired at multiple time points (0.5, 3, and 18–24 h after injection) after ^90^Y-FAPI-46 application. Data from at least 2 time points were necessary to determine lesion dose. Tumor and organ dosimetry was assessed by analyzing the respective regions of interest in the PET images, from which the pharmacokinetic behavior was fitted to monoexponential functions. Images were acquired in a Siemens mCT or Biograph Vision scanner, following an optimized protocol for quantification (16). PET quantification accuracy was validated in a National Electrical Manufacturers Association phantom, being considered most favorable when scanned in a silicon photomultiplier PET/CT scanner. Maximum liver and lung doses were assessed individually on the basis of minimum measurable ^90^Y-FAPI-46 uptake in prior PET phantom studies. We considered the number of disintegrations that would take place in the organ, assuming the minimum detectable activity concentration of 100 kBq/mL and the pharmacokinetics observed in blood dosimetry at the standard organ volumetry stated in the OLINDA.

### Outcomes and Statistical Analysis

Toxicity was recorded as per the Common Terminology Criteria for Adverse Events (version 5.0). Clinical, laboratory, and imaging follow-up was performed as per clinical routine, with laboratory and clinical visits every 2–4 wk and imaging within 1–2 mo. Imaging response was defined as per RECIST (version 1.1) for CT and PERCIST for ^18^F-FDG PET/CT (17*,*^18^). Disease control was defined as complete (metabolic) response, partial (metabolic) response, or stable (metabolic) disease. All patients received baseline imaging with ^18^F-FDG PET/CT to rule out sites of discordant disease. ^18^F-FDG PET/CT was performed 2 wk after the first cycle in 7 patients (78%) (Supplemental Figs. 1–9; supplemental materials are available at http://jnm.snmjournals.org). For overall response rate, response was defined as complete (metabolic) response or partial (metabolic) response. Descriptive statistics were used to present data; median and IQR were used for continuous measures, and absolute number and percentage were used for categoric data. No statistical tests were used for this study. All statistical analysis was performed using R statistics (version 3.4.1, www.r-project.org).

## RESULTS

### Patient Characteristics

Nine patients either with metastatic soft-tissue or bone sarcoma (*n* = 6) or with pancreatic cancer (*n* = 3) were treated between June 2020 and March 2021 (Table 1). The median age was 57 y (IQR, 55–62 y). At baseline, most patients had a median of 6 (IQR, 2–6.5) previous systemic treatment lines (Table 1) and were progressive during their last regimen. The Eastern Cooperative Oncology Group score for most patients was greater than or equal to 2 (*n* = 6; 67%), and only 3 patients had an Eastern Cooperative Oncology Group score of 1 at baseline (Table 1).

**TABLE 1.** Patient Characteristics

Patient no.	Age (y)	Sex	Histology	Tumor sites (primary and metastatic)	Eastern Cooperative Oncology group	No. of previous systemic therapies	Concomitant therapy	Subsequent therapy	^68^Ga-FAPI-46 (SUV_max_ baseline)	Status	Follow-up (d)
1	22	Male	Osteosarcoma	Lung, heart, lymph nodes	2	7	—	—	12.1	Dead	24
2	66	Male	Chordoma	Bone, soft tissue, liver, lung, lymph nodes	3	2	—	Nivolumab	22.3	Dead	67
3	54	Female	Fibrosarcoma	Lung, lymph nodes, pancreas, bone	1	6	—	—	18.3	Follow-up	100
4	57	Female	PDAC	Liver, lung, lymph nodes, bone	3	2	—	Cisplatin	14.9	Dead	57
5	61	Female	PDAC	Pancreas, liver, lung, lymph nodes, bone	2	9	Trametinib	—	19.4	Dead	41
6	56	Female	PDAC	Pancreas, liver, lung, lymph nodes, kidney	2	6		—	16.5	Dead	105
7	63	Female	GNET	Lung, liver, lymph nodes, bone, soft tissue	1	3	—	Nivolumab	16.1	Follow-up	44
8	61	Male	Conventional chondrosarcoma	Lung, lymph nodes, pancreas, bone	2	1	—	—	16.7	Follow-up	36
9	56	Male	Spindle cell sarcoma	Kidney, liver, lung pleura	1	6	—	—	28	Follow-up	36

PDAC = pancreatic ductal adenocarcinoma; GNET = gastrointestinal neuroectodermal tumor.

### Treatment and Dosimetry

Patients received a median dose of 3.8 GBq (IQR, 3.25–5.40 GBq) for the first cycle and 7.4 GBq (IQR, 7.3–7.5 GBq) for any subsequent cycle. Patient 3 received 3 cycles of ^90^Y-FAPI-46 with a cumulative activity of 18.3 GBq. Patients 8 and 9 each have received 2 cycles of ^90^Y-FAPI-46 for a total of 11.2 and 10.0 GBq, respectively. All other patients (*n* = 6) stopped treatment after the first cycle because of lack of focal ^90^Y-FAPI-46 uptake based on posttreatment ^90^Y-FAPI-46 scintigraphy in the tumor after the first cycle (*n* = 2) or rapid deterioration or death before the second cycle (*n* = 4).

Median renal absorbed dose was 0.52 Gy/GBq (IQR, 0.41–0.65 Gy/GBq; *n* = 4) per cycle. A median bone marrow absorbed dose of 0.04 Gy/GBq (IQR, 0.03–0.06 Gy/GBq; *n* = 5) was observed over all cycles. Liver and lung dosimetry was considered only for those patients on whom bone marrow dosimetry was performed. The maximum observed dose in liver and lung was less than or equal to 0.26 Gy/GBq, based on the assumptions presented in the methodology section.

**FIGURE 2.**
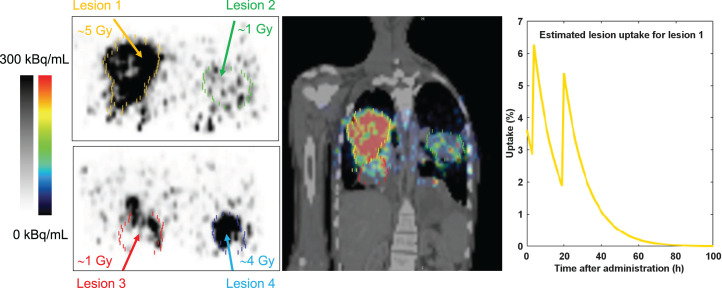
Posttreatment ^90^Y-FAPI-46 PET images 4 h after injection with corresponding absorbed dose estimates for 4 lesions in patient 2.

Lesion dosimetry was available for 9 lesions in 6 patients, exemplarily shown for patient 2 (Fig. 2). Median tumor effective half-life was 8.7 h (range, 5.5−18 h). Median dose absorbed by tumor lesions after the first cycle was 1.28 Gy/GBq (IQR, 0.83–1.71 Gy/GBq) per cycle for target lesions and 0.95 Gy/GBq (IQR, 0.74–1.32 Gy/GBq) for secondary lesions. The highest doses were observed in patients 6 (1.37 Gy/GBq), 3 (1.23 Gy/GBq), and 9 (2.28 Gy/GBq). For subsequent cycles in patients 3 and 9, a median lesion dose of 1.28 and 2.04 Gy/GBq per cycle was measured, respectively. Table 2 outlines the dosimetry results.

**TABLE 2.** ^90^Y-FAPI-46 Administered Activity and Absorbed Doses Per Cycle

Patient no.	Cycle no.	Activity (GBq)	Radiation dose (Gy/GBq)				
			Tumor lesion 1	Tumor lesion 2	Kidney	Liver and lung*	Bone marrow
1	1	7.1	0.74	0.63	—	—	—
2	1	7.0	—	—	—	—	—
3	1	3.5	1.23	1.23	0.75	<0.18	0.06
	2	7.3	1.28	0.95	0.41	<0.19	0.04
	3	7.5	1.47	1.35	0.61	<0.15	0.04
4	1	3.8	—	—	—	—	—
5	1	3.8	—	—	—	<0.16	0.06
6	1	3.0	1.37	—	—	—	—
7	1	3.5	0.91	0.84	0.52	<0.16	0.03
8	1	3.8	0.49	—	0.11	<0.26	0.08
	2	7.4	—	—	—	—	0.08
9	1	2.6	2.28	—	0.65	<0.21	0.04
	2	7.4	1.79	—	0.45	<0.25	0.02
Median			1.28	0.95	0.52	<0.19	0.04
IQR			0.83–1.71	0.74–1.32	0.41–0.65	<0.16–0.24	0.04–0.07

* Estimation based on maximum detectable activity concentration and blood tracer kinetic.

### Adverse Events and Follow-up

**FIGURE 3.**
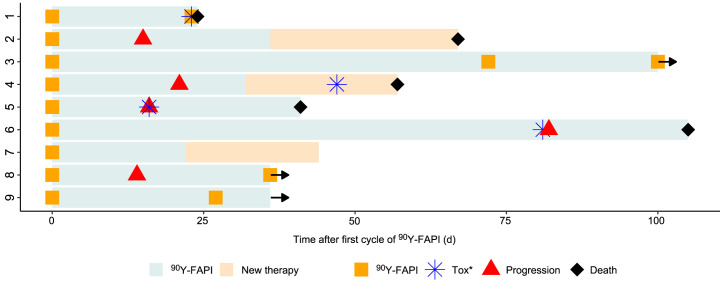
Swimmer plot of patients who received ^90^Y-FAPI-46. Arrows indicate patients continuing ^90^Y-FAPI-46 RLT at time of analysis. *Any new onset of toxicity greater than or equal to grade 3 according to Common Terminology Criteria for Adverse Events (version 5.0).

The median follow-up time was 44 d (IQR, 36–83.5 d). Three patients are still receiving RLT and had received 2 or 3 cycles at that point. Five patients died during follow-up. All 5 deaths were considered to be due to tumor progression and not related to ^90^Y-FAPI-46 (Tables 1 and 3). In patients with progression, the median time until progression or death was 18.5 d (IQR, 14.8–38.5 d). There were no acute or allergic reactions observed immediately after infusion of ^90^Y-FAPI-46. One patient, with advanced pulmonary metastasis and progressive intratumoral arteriovenous shunts, died because of acute respiratory failure attributed to tumor progression shortly after receiving his second cycle. Another patient developed a fever shortly after her first cycle, which was likely due to acute urinary tract infection and noncompliance with antibiotic medication. At baseline, 5 patients had one or more ongoing toxicities greater than or equal to grade 3. These were anemia (*n* = 2), increase of alkaline phosphatase (*n* = 1), or increase of γ-glutamyltransferase (*n* = 3) (Table 3). During follow-up, 4 patients showed new grade 3 or grade 4 laboratory toxicities (Table 3; Fig. 3). These 4 new adverse events were grade 3 thrombocytopenia (*n* = 4) possibly related to ^90^Y-FAPI-46 and were also in temporal relation to either tumor progression or initiation of other concomitant systemic therapy (Fig. 3). One patient showed new grade 3 anemia, and 2 patients showed new increases of hepatic or pancreatobiliary serum markers greater than or equal to grade 3 (Table 3). All 3 of these new adverse events were rated as disease progression, given that all 3 of these patients had pancreatic cancer (Fig. 3). A detailed course of the relevant laboratory parameters is shown in Supplemental Figure 10.

**TABLE 3.** Adverse Events After Onset of Treatment, Related or Unrelated

		Laboratory-based AEs																						
		Hematology								Kidney		Liver						Pancreatobiliary						
		WBCs		ANC		Hb		PLTs		sCr		T Bil		AST		ALT		GGT		ALP		Amylase		New G3/G4 AE (laboratory)
Patient no.	General	B	F	B	F	B	F	B	F	B	F	B	F	B	F	B	F	B	F	B	F	B	F	
1	Acute respiratory distress, tumor-related (G5)	—	—	—	—	G3	G2	—	G3	—	—	—	—	—	—	—	—	G2	G1	G1	—	—	—	Yes
2	Tumor pain (G2)	—	—	—	—	—	G1	—	G1	—	—	—	—	—	—	—	—	G1	G1	G1	G1	—	—	No
3	None	—	—	—	—	—	—	—	G1	—	—	—	—	—	—	—	—	G1	G1	G1	G1	—	—	No
4	Tumor progression, (G5)	G1	G2	—	G1	G3	G3	G1	G3	—	G2	—	G3	—	G1	—	—	G1	G3	G2	G2	—	—	Yes
5	Tumor progression (G5)	—	—	—	—	G1	G2	G1	G3	—	G1	—	G2	G2	G4	G1	G4	G3	G4	G3	G3	—	—	Yes
6	Pneumonia*, tumor progression (G5)	—	—	—	—	G1	G3	G1	G3	—	G2	—	G2	—	G2	—	—	G3	—	G1	G2	—	—	Yes
7	Fever, urinary tract infection*	—	—	—	—	G1	G2	—	—	—	—	—	—	—	—	—	—	—	—	—	—	—	—	No
8	None	—	—	—	—	G1	—	—	—	—	—	—	—	—	—	—	—	—	G1	G1	G1	—	—	No
9	None	—	—	—	—	G1	G1	—	—	—	—	—	—	—	—	—	—	G3	G3	G2	G2	—	—	No
	Any new AE (%)	1 (11%)		1 (11%)		3 (33%)		6 (67%)		3 (33%)		3 (33%)		3 (33%)		1 (11%)		3 (33%)		1 (11%)		—		
	Any new G3/G4 AE (%)	—		—		1 (11%)		4 (44%)		—		1 (11%)		1 (11%)		1 (11%)		2 (22%)		—		—		

* Relation to ^90^Y-FAPI-46 was ruled out.

AE = adverse event; WBCs = white blood cells; ANC = absolute neutrophil count; Hb = hemoglobin; PLTs = platelets (thrombocytes); sCr = serum creatinine; T Bil = total bilirubin; AST = aspartate transaminase; ALT = alanine transaminase; GGT = γ-glutamyltransferase; ALP = alkaline phosphatase; B = baseline; F = follow-up.

G grade is defined as per CTCAE, version 5.0.

### Response Evaluation

Radiologic response as per RECIST (version 1.1) was available for 8 patients. The median time between imaging and the first cycle of ^90^Y-FAPI-46 was 16 d (IQR, 15–41 d). Disease control (stable disease) was noted in 4 of 8 patients (50%). No responses had been observed by the time of analysis. However, patient 3 had marked regression of a target lesion (−28%; Supplemental Fig. 3) after the first cycle with 3.5 GBq. Metabolic response as per PERCIST (version 1.0) was available for 7 patients. Disease control was noted in 2 of 7 patients (29%), consisting of stable metabolic disease in one patient (14%; Supplemental Fig. 3) and partial metabolic response in the other (14%; Supplemental Fig. 9). Radiologic responses are outlined in Table 4.

**TABLE 4.** Radiologic and Metabolic Best Overall Response

Patient no.	CT target response	CT nontarget response	RECIST response	PET target response	PET nontarget response	PERCIST response	SUV_max_^18^F-FDG baseline	SUV_max_^18^F-FDG follow-up
1	SD	SD	SD	PMR	SMD	PMD	14.8	21.8 (+47%)
2	PD	SD	PD	PMD	PMD	PMD	28.6	22.3 (−22%)
3	SD	SD	SD	SMD	SMD	SMD	6.5	4.9 (−25%)
4	PD	PD	PD	SMD	PMD	PMD	5.1	3.8 (−26%)
5	PD	PD	PD	SMD	PMD	PMD	18.9	17.2 (−9%)
6	SD	SD	SD	—	—	—	6.1	—
7	—	—	—	—	—	—	14.3	—
8	SD	PD	PD	PMD	SMD	PMD	12.5	13.3 (+6.4%)
9	SD	SD	SD	PMR	SMD	PMR	18	10.1 (−44%)
DCR (%)			4/8 (50%)			2/7 (29%)		
ORR (%)			0/8 (0%)			1/7 (14%)		

SD = stable disease; PMR = partial metabolic response; SMD = stable metabolic disease; PMD = progressive metabolic disease; PD = progressive disease; DCR = disease control rate; ORR = overall response rate.

## DISCUSSION

We here report the first case series of patients with advanced-stage solid tumors treated with ^90^Y-FAPI-46 RLT. Repeated ^90^Y-FAPI-46 applications with individual dosimetry were used to ensure the safety of each patient and a maximum likelihood of treatment effect. For treatment initiation, patients had to have high uptake on ^68^Ga-FAPI-46 PET in most tumor lesions, and for treatment continuation, patient had to have focal uptake on the first posttreatment ^90^Y-FAPI-46 bremsstrahlung scintigraphy (Fig. 1; Supplemental Figs. 1–9). Patients had exhausted all available on-label or evidence-based treatment options, and the most prevalent Eastern Cooperative Oncology Group score was 2 or higher. Treatment with ^90^Y-FAPI-46 was offered under compassionate use with the intent of achieving antitumor effect with manageable toxicity. On the basis of the biodistribution observed on ^68^Ga-FAPI-46, PET RLT using ^90^Y-FAPI-46 was expected to deliver therapeutic radiation doses to the tumor while sparing organs at risk (4*,*^11^). Acute toxicities or immediate (e.g., allergic) reactions to RLT were not observed. During follow-up, adverse events began in almost all patients. However, only a small proportion was attributed to ^90^Y-FAPI-46, given that most adverse events occurred after tumor progression or the switch of systemic therapy (Fig. 3). Additionally, we noted that toxicity in 1 patient who had received multiple RLT cycles with a cumulative activity of 18.3 GBq was limited to G1 thrombocytopenia. Ultimately, randomized trials on patients with symptomatic disease are needed for more detailed assessment of toxicity. Data from previous randomized trials evaluating ^177^Lu-PSMA-617 or ^177^Lu-DOTATATE identified hematotoxicity, especially thrombocytopenia, as a relevant (i.e., frequently occurring as grade 3/4) side effect (19*,*^20^). On the basis of our data, we expect a similar toxicity profile for ^90^Y-FAPI-46. Therefore, repeated cycles of ^90^Y-FAPI-46 RLT seem feasible, because the doses absorbed by the kidneys, bone marrow, liver, and lungs were low and comparable to those of other small-ligand ^90^Y therapies (21). In our cohort, 3 patients received multiple cycles with a maximum cumulative activity of up to 18.3 GBq.

When all other available therapeutic options fail, achieving disease control is the primary goal for a novel therapy. Previously, Kratochwil et al. reported on a patient with spindle cell soft-tissue sarcoma who had a long period of stable disease under FAPI-46 RLT (12). Although the follow-up time is still short, we observed radiographic disease control in about half the patients, along with signs of tumor response. Patient 3 experienced meaningful benefit in the form of stable disease for over 4 mo, with regression of a large pancreatic tumor mass. Patient 9 showed a partial metabolic response and achieved the highest lesion dose with 13.2 Gy during cycle 2. Patients 3, 8, and 9 had additional cycles pending at the time of analysis. Interestingly, 3 of the 4 patients with disease control were patients with soft-tissue (*n* = 2) or bone (*n* = 1) sarcoma. The fourth patient had pancreatic cancer and received concomitant treatment with the tyrosine kinase inhibitor afatinib, which was well tolerated, therefore indicating the potential feasibility of combination therapy. In the quest to provide the most efficacious therapy with acceptable toxicity, especially in nonresponders, 2 future strategies should be considered: first, a more intense treatment regimen (i.e., short intercycle intervals or higher activities) and, second, RLT drug combination therapy. FAP and cancer-associated fibroblasts are drivers of immune escape (22*,*^23^); therefore, immunotherapy might be a rationale companion for FAP-targeted RLT. Preclinical studies in several cancer types suggest a synergistic effect of FAP targeting and immunotherapy (24–27). Recently, a case report showed good tolerance of ^177^Lu-PSMA RLT in combination with pembrolizumab or sequentially after olaparib (28), which is currently being investigated in ongoing prospective phase 1/2 trials (NCT03874884, NCT03805594).

^90^Y-FAPI-46 has a shorter half-life and higher energy per decay than ^177^Lu-PSMA. Because of the short retention time in the tumor as described by Lindner et al. (14), ^90^Y-FAPI-46 seemed more suitable for achieving therapeutic radiation doses in a tumor. ^90^Y-FAPI-46 PET-based dosimetry has been successfully used for hepatic radioembolization dosimetry, after administration of ^90^Y-labeled spheres (29). Phantom studies suggest that recent developments in sensitivity and timing resolution for PET scanners could be advantageous for accurate ^90^Y quantification, (16) which could play a decisive role in the validation of ^90^Y-labeled therapeutic drugs.

This study comes with limitations. The low number of patients and absence of a predefined imaging follow-up protocol does not allow for definitive conclusions regarding therapeutic efficacy and toxicity of ^90^Y-FAPI-46. Further research to determine radiation dosimetry for ^90^Y-FAPI-46 is warranted, because quantification and subsequent dosimetry are limited by the decay characteristics of ^90^Y-FAPI-46 and the relatively low activity concentration in tissues. A low activity concentration combined with detector limits impairs accurate acquisition of the true lung and liver doses. However, the aim of this study was to report the initial clinical experience and to demonstrate the feasibility of ^90^Y-FAPI-46 RLT.

## CONCLUSION

FAP-targeted RLT with ^90^Y-FAPI-46 was well tolerated, with a low rate of attributable adverse events, including thrombocytopenia. We found low radiation doses to the kidney and bone marrow, suggesting the feasibility of repeated cycles of ^90^Y-FAPI-46. Although we observed the first signs of therapeutic efficacy, larger trials are needed to determine efficacy and the toxicity profile.

## Notes

KEY POINTS **QUESTION:** Is radionuclide therapy with ^90^Y-FAPI-46 feasible for patients with advanced-stage solid tumors, and what are the side effects and absorbed doses? **PERTINENT FINDINGS:** ^90^Y-FAPI-46 leads to therapeutic irradiation of tumor lesions, and the radiation exposure of critical organs is low. Further, we observed, after a short follow-up, a low rate of toxicities, including thrombocytopenia, attributed to ^90^Y-FAPI-46 in patients with advanced and symptomatic disease. **IMPLICATIONS FOR PATIENT CARE:** Radionuclide therapy with ^90^Y-FAPI-46 seems to be well tolerated, and repeated cycles are possible.
